# Supplementary material for: Genome-Wide Association Studies of Salt Tolerance at the Seed Germination Stage and Yield-Related Traits in Brassica napus L
Source: Int J Mol Sci. 2022 Dec 14;23(24):15892. doi: 10.3390/ijms232415892 (PMC9785822; doi:10.3390/ijms232415892)
Supplement: Supplementary file 1 [file ijms-23-15892-s001.zip › Supplementary Materials.pdf]

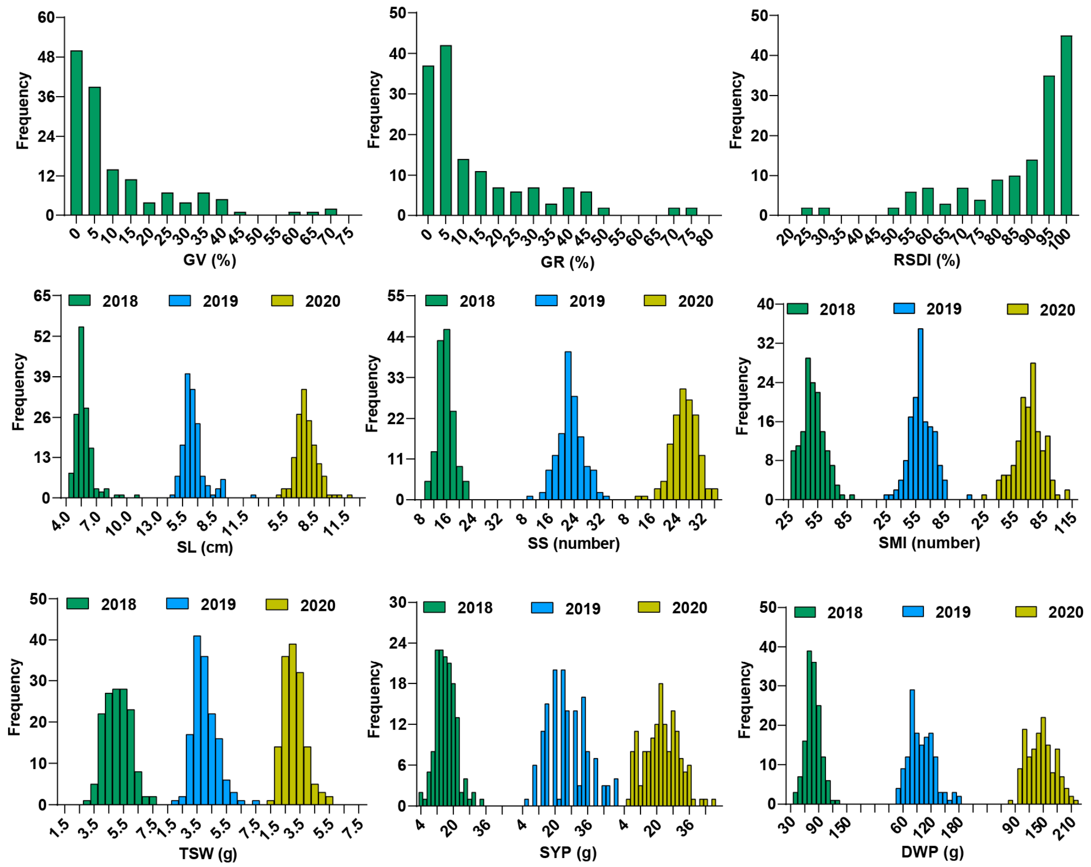


**Figure S1**. The frequency distribution of all traits for 146 rapeseed accessions. The x-axis indicates the ranges of phenotypic data for each trait; the y-axis indicates the number of individuals. SL, silique length; SMI, siliques on main inflorescence; SS, seeds per silique; TSW, thousand seeds weight; SYP, seed yield per plant; DWP, dry weight per plant; GV, germination vigor; GR, germination rate; RSDI, relative salt damage index.

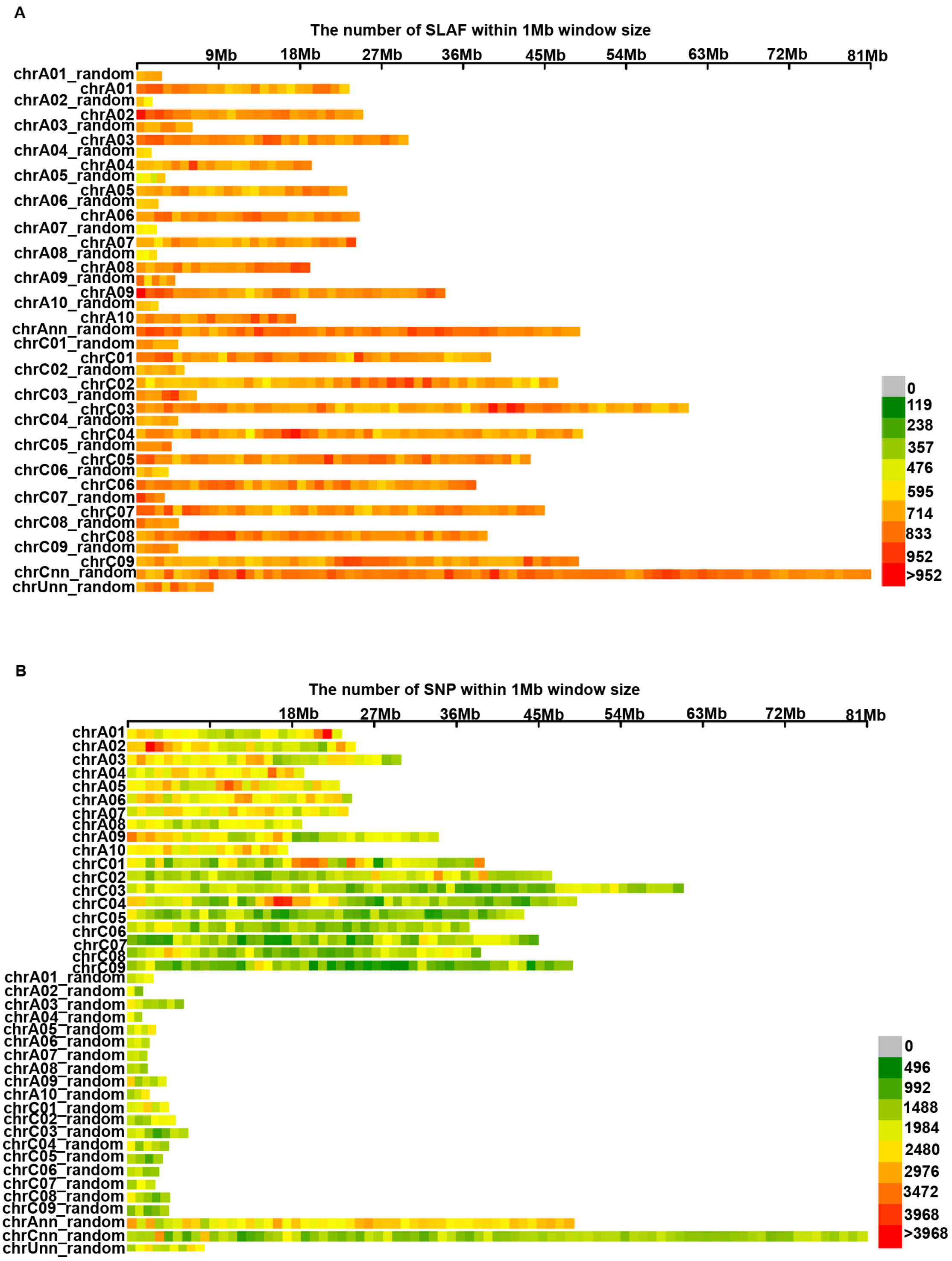


**Figure S2**. Distribution of the specific-locus amplified fragment (A) and SNPs (B) on the 19 rapeseed chromosomes. Each row represents one chromosome and the colors indicate the number of SLAF tags or SNPs within a Mb window.

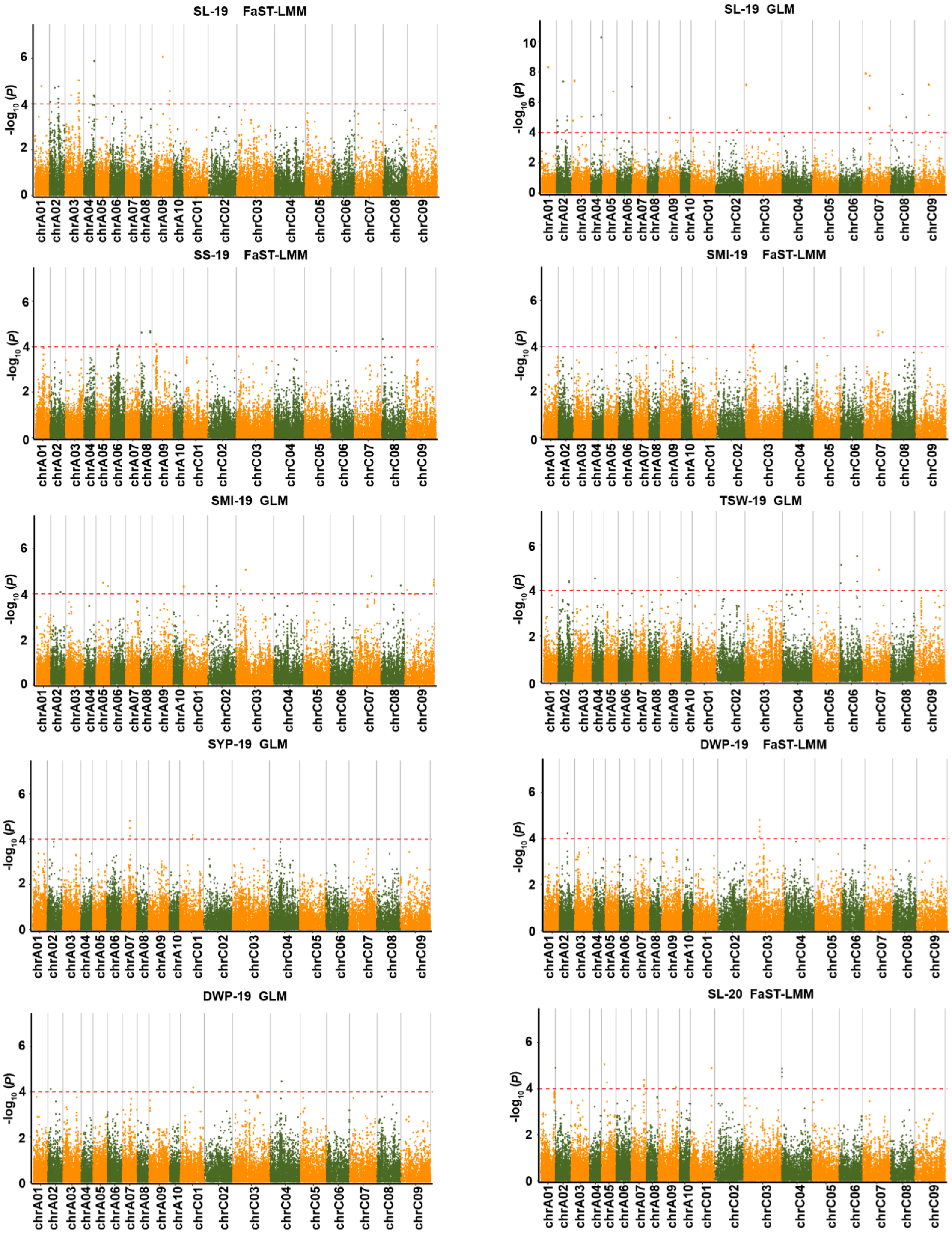
SL-19 FaST-LMM
SL-19 GLM
SS-19 FaST-LMM
SMI-19 FaST-LMM
SMI-19 GLM
TSW-19 GLM
SYP-19 GLM
DWP-19 FaST-LMM
DWP-19 GLM
SL-20 FaST-LMM
$-\log_{10}(P)$
chrA01 chrA02 chrA03 chrA04 chrA05 chrA06 chrA07 chrA08 chrA09 chrA10 chrC01 chrC02 chrC03 chrC04 chrC05 chrC06 chrC07 chrC08 chrC09

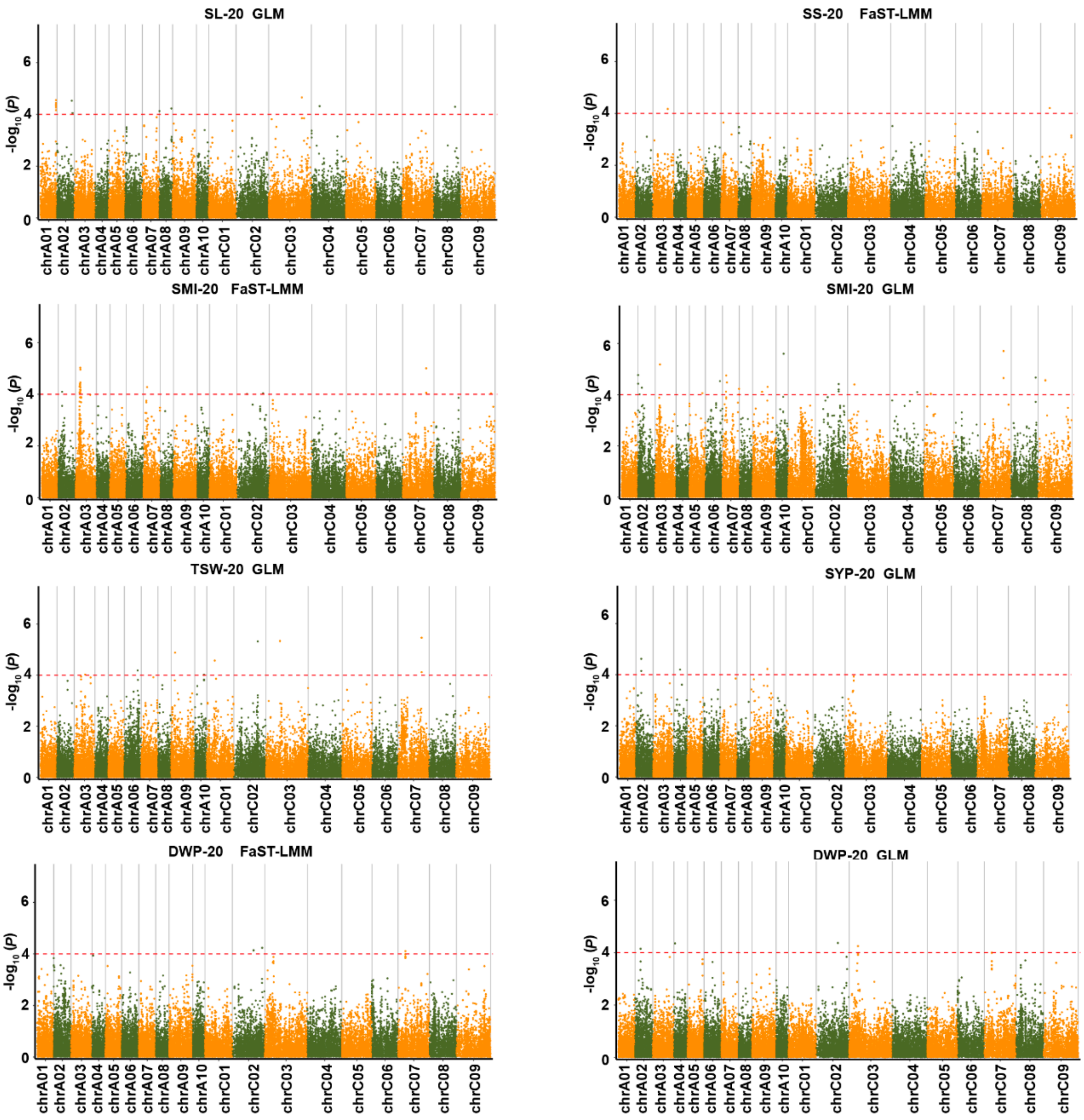


**Figure S3**. Manhattan plots from association analysis of six yield-related traits using the optimal statistical models in two year (2019, 2020). The X-axis indicates the nineteen chromosomes and the Y-axis represents -log10 (P) values of the SNP marker. The red dashed line represents the significant threshold (P < 0.0001). GLM, general linear model; FaST-LMM, factored spectrally transformed linear mixed model. SL, silique length; SMI, siliques on main inflorescence; SS, seeds per silique; TSW, thousand seeds weight; SYP, seed yield per plant; DWP, dry weight per plant.
